# RETRACTED: Zhang et al. SrFe_x_Ni_1−x_O_3−δ_ Perovskites Coated on Ti Anodes and Their Electrocatalytic Properties for Cleaning Nitrogenous Wastewater. *Materials* 2019, *12*, 511

**DOI:** 10.3390/ma16206818

**Published:** 2023-10-23

**Authors:** Yuqing Zhang, Zilu Jin, Lijun Chen, Jiaqi Wang

**Affiliations:** School of Chemical Engineering and Technology, Tianjin University, Tianjin 300350, China

The journal retracts the article “SrFe_x_Ni_1−x_O_3−δ_ Perovskites Coated on Ti Anodes and Their Electrocatalytic Properties for Cleaning Nitrogenous Wastewater” [1], cited above. 

Following publication, concerns were brought to the attention of the journal regarding the XRD data.

Adhering to our complaints procedure, an investigation was conducted. The authors were not able to provide the raw data and the Editor-in-Chief and editorial board no longer have confidence in the reported results. The article is therefore retracted.

This retraction was approved by the Editor-in-Chief of the journal *Materials*.

The authors did not agree to this retraction.
